# Social robots in adult psychiatry: a summary of utilisation and impact

**DOI:** 10.3389/fpsyt.2025.1506776

**Published:** 2025-02-11

**Authors:** Maja Kling, Alfred Haeussl, Nina Dalkner, Frederike T. Fellendorf, Melanie Lenger, Alexander Finner, Julia Ilic, Irina S. Smolak, Lena Stojec, Ina Zwigl, Eva Z. Reininghaus

**Affiliations:** Division of Psychiatry and Psychotherapeutic Medicine, Medical University of Graz, Graz, Austria

**Keywords:** social robot, mental health, schizophrenia, autism spectrum disorder, intellectual disability, digital health

## Abstract

Social robots are increasingly becoming more prevalent in healthcare, including nursing, geriatric care, and treatment for children on the autism spectrum. Their assistance is believed to hold promise in mitigating the effects of staffing shortages and enhancing current mental health treatment. Nevertheless, the application of social robotics in psychiatry remains restricted and controversial. This scoping review aims to provide an overview of the literature on social robots in adult psychiatry concerning their use, effects, and acceptability. We conducted a literature search, including PubMed and PsycINFO, to identify literature on robot interventions for adult psychiatric patients. Methodological quality was assessed using the ‘Mixed Methods Appraisal Tool’. Usage and target variables were unique to every included original study (N = 7) and suggested a wide range of possible implications for patient treatment and care. Social robots were used to reduce symptoms, improve functioning, and gain insights into characteristic features of specific mental health conditions. The included studies were concerned with the following diagnoses: schizophrenia (N = 3), autism spectrum disorder (N = 2), and intellectual disability (N = 2). The sample sizes were too small to generalise the outcome, but overall trends showed some positive effects on the selected symptoms. Observations and participant feedback suggested high acceptance and enjoyment of the users. Although the evidence regarding the benefits of robotic interventions in adult psychiatry is still low, it suffices to assume that investing in larger, randomised, and controlled trials is worthwhile and promising.

**Systematic review registration:**

asprediced.org, identifier 128766.

## 1 Introduction

According to a recent World Health Organization (WHO) report on the health and care workforce, all countries in the European Region face severe challenges in covering their populations’ health and care demands (1). Using Austria as an example, it is projected that by 2030, approximately 34.000 additional nurses, nursing assistants, and social workers will be required compared to 2017 (2). A 2017 survey revealed that 23% of the Austrian population experienced psychiatric symptoms or illnesses within a year, with around 14% requiring psychotherapeutic treatment (3). However, only 2.8% of Austrians currently receive publicly financed support to access psychotherapy sessions (4). One proposed strategy to address the disparity between the demand for mental health care services and existing human resources is the expanded utilisation of digital tools (1). The deployment of social robots in the field of mental health is one possible way to do so.

**Figure 1:**
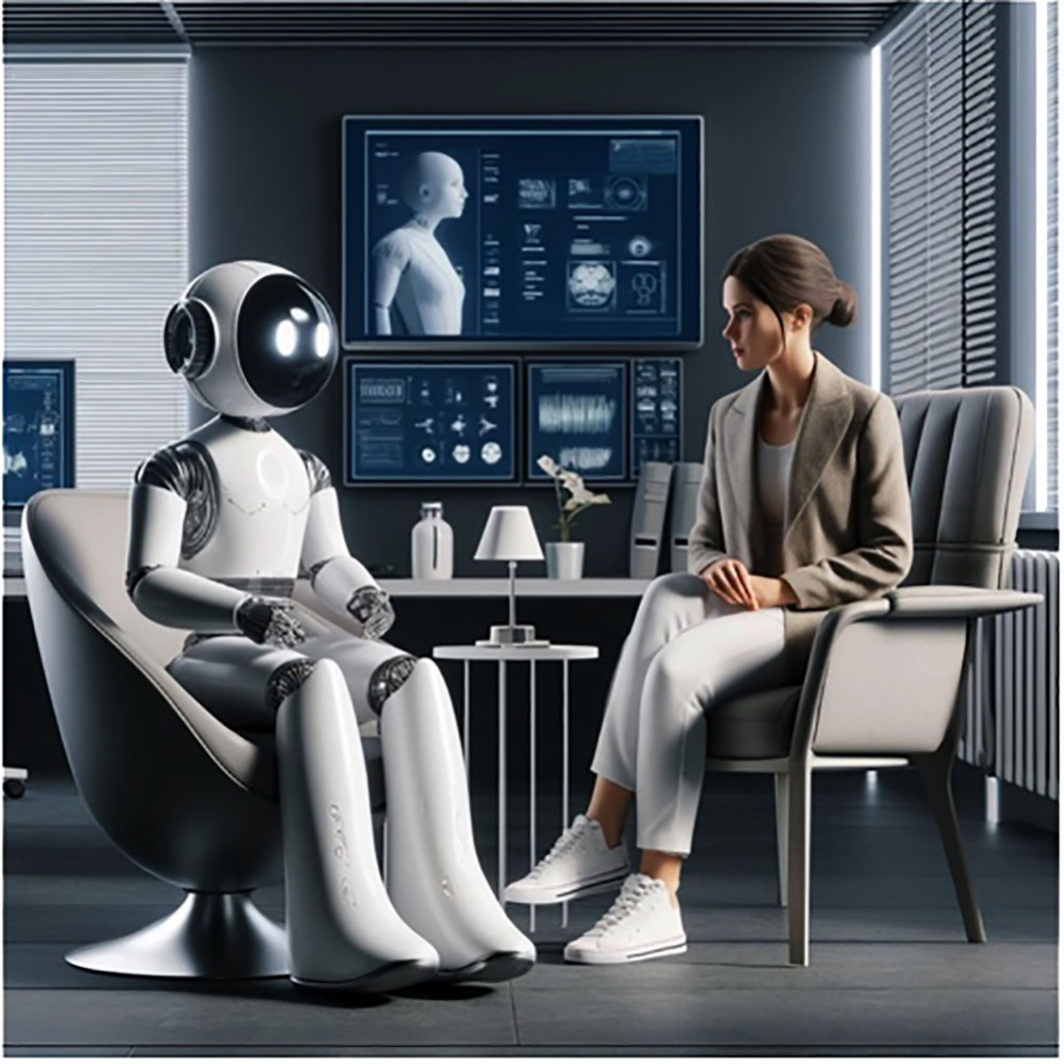
Illustraion created by OpenAI’s DALL-E 2, a generative AI model specialised in creating images based on textual descriptions.

No universal definition of social robots exists, particularly regarding what makes the robot ‘social’ (5). Duffy (6) proposed to define social robots as a physical entity embodied in a complex, dynamic, and social environment sufficiently empowered to behave in a manner conducive to their own goals and those of their community. Mejia and Kajikawa (7) found that social robotics research often focuses on ‘robots as social partners,’ which includes robots as companions, teachers for children, and assistants for older adults. Accordingly, a robot must be embodied in a humanoid (**Figure 1**) or zoomorphic form, capable of direct physical interaction with humans through verbal or nonverbal means to be considered ‘social’. This interaction may include engaging in dialogue, producing sounds, displaying emotions, responding to touch, and executing purposeful movements.

Digital applications, such as social robots, could not only play a key role in addressing the growing demands of healthcare but also offer intrinsic therapeutic benefits that complement existing therapy approaches. For instance, discussing sensitive topics with a robot may reduce feelings of judgment, stigma, or the fear of ‘wasting someone’s time’ (8). For individuals with social anxiety, robotic therapists could help lower anticipatory anxiety and improve treatment adherence, for example by simulating social situations as a form of rehearsal in a safe space (9).

In healthcare, a large body of research has focused on social robot interventions for people with dementia (10). In geriatric care, social robots have been successful in alleviating depressive symptoms and improving feelings of loneliness and overall quality of life (11). They have been used as companions and therapy mediators (12).

Research has also focused on children with Autism Spectrum Disorder (ASD) (13). Robot-assisted therapy shows promise in helping children with ASD develop social skills, such as collaborative play behaviour, verbal communication, and visual attention (14). However, children may respond differently to robots due to factors like limited critical reflection, heightened susceptibility to interference, and distinct cognitive abilities (15). As a result, research findings in children may not directly apply to adults and should be evaluated separately.

In contrast to these relatively well-studied groups, there is limited robust research on social robot interventions for adults with mental disorders (16).

Further, our initial literature search revealed a research gap regarding facility-based psychiatric care. This paper focuses on synthesizing the limited yet impactful body of research on this niche subject, situating it within the broader context of psychological and psychiatric care.

Thus, we increase accessibility of existing data for researchers with a specific focus on this topic, while also deriving general recommendations that may hold relevance and applicability across various forms of psychiatric care delivery.

The following questions are addressed:

1. Which target variables were investigated during social robot interventions?
2. What were the pertinent effects on these target variables? What has been observed?
3. How are interventions involving social robots received, and what are the participants’ perceptions?

This scoping review aims to facilitate and advance research in this emerging field by summarising preliminary findings and eventually ensuring the latest therapeutic innovations are accessible to psychiatric patients.

## 2 Methods

### 2.1 Search strategy

The protocol for this scoping review adhered to the PRISMA-ScR (Preferred Reporting Items for Systematic Reviews and Meta-Analyses Extension for Scoping Reviews) guidelines.

A review protocol was registered on asprediced.org (Registration Number 128766). The databases PubMed and PsycINFO were systematically searched using predefined keywords: (“social* robot*” OR “social* assistive robot*” OR “companion robot*” AND psych*). Additionally, reference lists of relevant articles were screened for further studies. Only published research papers, excluding conference papers, were considered for this review. No restrictions were placed on the publication year to ensure a comprehensive evaluation of the available evidence.

The most recent database search was conducted on March 6, 2024. Results were imported into the screening tool Rayyan, where two reviewers (M.K. and A.H.) independently screened article titles. Any discrepancies were resolved through discussion and mutual agreement.

Given the heterogeneity of analysed parameters and outcome measures, a narrative approach was selected to summarise target variables, effects, observations, and acceptability of the interventions.

### 2.2 Selection criteria

To be eligible for inclusion, studies were required to (1) involve a physically embodied social robot; (2) investigate adults aged 18 or older diagnosed with a mental health disorder; (3) be conducted within the setting of a mental health institution; (4) present original research on the effects or acceptability of the robot; and (5) be written in English.

To our knowledge, no official definition of a ‘mental health institution’ is available. For this review, it was defined as ‘any hospital, institution, clinic, evaluation facility, mental health centre, or part thereof, which is used primarily for the care or treatment of persons with mental illnesses’ (17).

All participants were required to have a confirmed mental health condition recognised by the International Statistical Classification of Diseases and Related Health Problems (ICD-10) of the WHO 2019 register.

Studies were excluded if they solely described the development of an experimental setup or robot design, or if they utilised a telepresence robot with no social interaction capabilities beyond video calls.

### 2.3 Assessment of quality

Quality approval of the included studies was conducted using the Mixed Methods Appraisal Tool (MMAT), version 2018 (18). Designed for systematic mixed-studies reviews, the MMAT allows for the evaluation of methodological quality across five study categories: qualitative research, randomised controlled trials, non-randomised studies, quantitative descriptive studies, and mixed-methods studies.

The appraisal process begins with two screening questions, both of which must be answered with ‘Yes’ to confirm the study’s eligibility for MMAT evaluation. The appropriate category is then selected, and each of the five criteria within that category is rated using ‘Yes’, ‘No’, or ‘Can’t tell’. Following best practices, we provided a detailed presentation of the ratings for each criterion to report the quality of the included studies in a nuanced manner, rather than calculating an overall score (18).

The appraisal aimed not to exclude studies based on poor quality but to offer a descriptive overview of methodological strengths and weaknesses and to identify research gaps. Two researchers independently conducted the appraisals to ensure accuracy and reliability.

## 3 Results

### 3.1 Included studies

**Figure 2:**
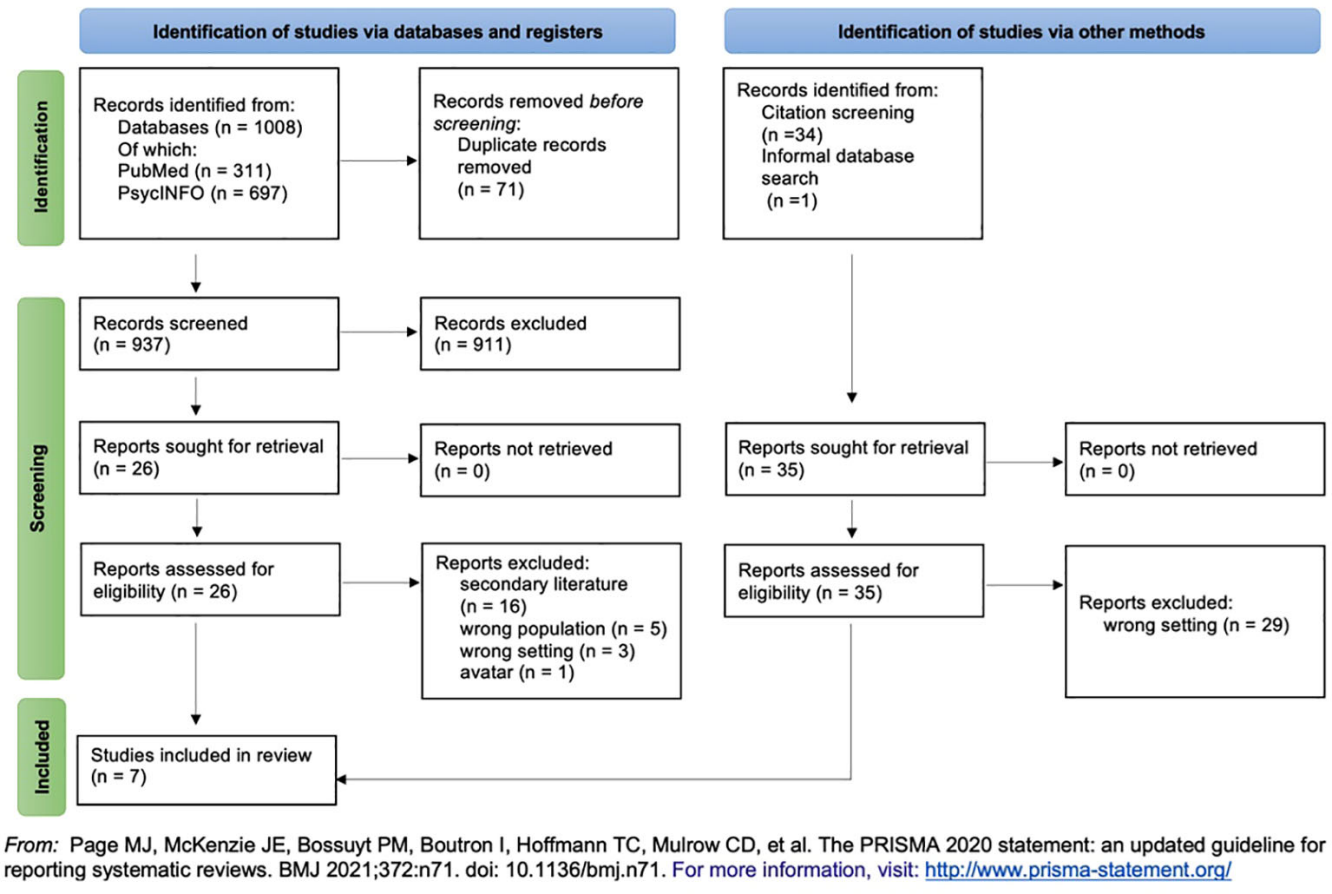
Prisma flow chart.

**Figure 2** illustrates the selection process for the reviewed records, following the PRISMA flow diagram (19).

After deleting duplicates, 911 publications were excluded based on their titles and abstracts. The two reviewers conducted a full-text screening on the remaining 26 eligible papers. During this process, citation screening of reference lists identified an additional 34 records for full-text screening. One further paper was included through an initial informal web search.

Ultimately, seven publications met all inclusion criteria and were included in the analysis.

- Three studies focused on individuals with schizophrenia (20–22).
- Two studies examined people with ASD (23, 24).
- Two studies investigated effects on individuals with Intellectual Disabilities (ID) (25, 26).

### 3.2 Target variables, effects, and observations

**Table 1** provides a detailed overview of the included studies (N =7), covering sample sizes and characteristics, the robots used, study settings, designs, target variables, and methods of measuring intervention acceptance.

**Table 1:** Characteristics, target variables, acceptance measures of included studies (N = 7).

Schizophrenia						
Author (Year), Country	Sample size and sample description	Robot	Setting	Study design	Target variables & Acceptance (A)	Results
Betriana et al. (2022) (20), Japan	N = 4 (N = 2 SZ patients, N = 2 mentally healthy controls)	Pepper	Ward of psychiatric hospital	Case reports	-characteristics of interactive communication between Pepper and patients with SZ compared to healthy participants A: Interviews of participants	-two-way dialogue possible -issues regarding eye contact -differences in perception -enjoyment of users
Cohen et al. (2017) (21), France	N = 44 (N = 22 SZ patients, N = 22 healthy controls)	iCub	University Department of Adult Psychiatry	Controlled trial	-influence of social feedback generated by a humanoid robot on motor coordination of SZ patients compared to healthy controls A: -	-patients were impaired in ability to synchronise with robot partner
Narita et al. (2016) (22), Japan	N = 3 SZ patients	AIBO	Ward of psychiatric hospital	Pilot study	-changes in PANSS and A-state STAI scores pre-/post-intervention: AAT (robot dog) A: Quotes of participants	-PANSS scores improved in some categories -STAI scores increased in all patients -positive tone of quotes
Autism Spectrum Disorder						
Author (Year), Country	Sample size and sample description	Robot	Setting	Study design	Target variables & Acceptance (A)	Results
Kumazaki et al. (2017) (23), Japan	N = 15 young adults with ASD (N = 7 in robot group, N = 8 in control group)	Actroid-F	Medical centre for developmental disorders	RCT	-feasibility and preliminary effectivity of an android robot-mediated mock job interview training in terms of both bolstering self-confidence and reducing biological levels of stress (SCL) in comparison to psychoeducation A: Informal observations	-robot-mediated systems may be feasible and effective to improve self-confidence and enhance performance with potential for transferability to real life settings -good acceptance
Kumazaki et al. (2019) (24), Japan	N = 29 young adults with ASD (N = 13 in robot group, N = 16 in control group)	Actroid-F	Institute for developmental disorders	RCT	-the acquirement of nonverbal communication, self-confidence, changes in biological levels of stress (SCL) undergoing a job interview training using an android robot A: Question: Do you want to receive this intervention again?	-improvement in various communication skills -improved biological stress reaction -positive trend in self-confidence ratings -higher satisfaction in intervention group
Intellectual Disability						
Author (Year), Country	Sample size and sample description	Robot	Setting	Study design	Target variables & Acceptance (A)	Results
Shukla et al. (2019) (25), Spain	N = 6 persons with moderate to severe ID	NAO	Residential mental health care institution	Case reports	- the response of robot interactions among individuals with ID, measuring engagement rates and disability behaviour during the robot interaction compared to normal life situations A: -	-interactive activities were more appealing -reduction of disability behaviour -necessity for customised applications
Wagemaker et al. (2017) (26), Netherlands	N = 5 persons with moderate to severe ID	Paro	Residential mental health care institution	Case reports	- the effectivity of Paro in adults with moderate to severe ID on mood and alertness (YCSRS, AOL) compared to plush seal A: Informal observations	-only one participant improved in chosen outcome measures -positive interactions were observed -mild anxiety in some participants -Paro attracted more attention

Description: AAT, Animal Assisted Therapy; ASD, Autism Spectrum Disorder; AOL, Alertness Observation List; ID, Intellectual Disability; PANSS, Positive and Negative Symptom Scale; RCT, Randomised Controlled Trial; SCL, Salvia cortisol level; STAI, State-Trait Anxiety Inventory; SZ, Schizophrenia and YCSRS, Young Child Session Rating Scale.

#### 3.2.1 Schizophrenia

Betriana et al. (20) employed intentional observations and interviews to explore the ‘characteristics of interactive communication’ between the humanoid robot ‘Pepper’ (27) and two patients with schizophrenia compared to two mentally healthy participants. The study aimed to evaluate Pepper’s ability to engage with multiple individuals simultaneously and to identify variations in participant responses. Following the interactions, participants were asked about their experiences, including their feelings and impressions. Data collection was guided by the ‘Intentional Observational Clinical Research Design (IOCRD)’ (28).

Each group of two participants talked with Pepper for 20-30 minutes.

The researchers observed that both schizophrenia patients and healthy controls enjoyed the interactions. A two-way dialogue was possible, and an intentional conversation was initiated. All participants answered Pepper’s questions appropriately and asked some questions themselves. Some requested entertainment, such as Pepper singing songs, but Pepper was unable to comply.

Key differences emerged between the groups. Patients with schizophrenia maintained consistent eye contact with Pepper, despite its inability to reciprocate, whereas the healthy controls expressed unease due to this lack of eye contact. Moreover, schizophrenia patients perceived Pepper as autonomously initiating conversations, a perception not shared by the healthy participants. Only the healthy participants noticed delays in Pepper’s response times, highlighting a disparity in sensitivity to the robot’s limitations.

Cohen et al. (21) conducted a controlled trial to analyse the impact of social feedback provided by the humanoid robot ‘iCub’ (29) on the motor coordination in 22 schizophrenia patients compared to 22 healthy controls. The robot’s and the participant’s alliance quality were assessed using an imitation task called the ‘mirror game’, were participants were instructed to follow the robot’s hand movements. Positive feedback was delivered whenever participants improved in synchrony metrics, including position error, velocity error, and sum of velocities compared to the previous five seconds. Three forms of feedback were provided in random order, each for 60 seconds. Feedback types included a smiling robot (social feedback), a plus symbol displayed on a tablet affixed to iCub’s face (non-social feedback) and a neutral robot expression (no feedback).

Participants were evaluated using the Neurological Soft Signs Scale (NSS) (30) and the Trail Making Test (TMT) parts A and B (31) as part of the cognitive assessment. Patients with schizophrenia also completed the Positive and Negative Syndrome Scale (PANSS) (32). It measures positive and negative symptoms of schizophrenia. Synchrony was quantified using the ‘Socio-Motor Coordination index (SMCi)’ (21).

The findings revealed that schizophrenia patients exhibited lower synchrony with the robot than healthy controls, regardless of feedback type. While social feedback increased SMCi scores in healthy controls, this effect was less pronounced in the schizophrenia group. Regression analysis showed that impaired cognitive flexibility, as measured by TMT, negatively influenced synchrony in patients. The study concluded that schizophrenia patients demonstrate reduced ability to synchronize with a robot in simple motor tasks, extending prior evidence of coordination challenges to human-robot interaction.

Narita et al. (22) examined changes in PANSS and A-state STAI (anxiety) (33) scores in three schizophrenia patients before and after engaging in Animal Assisted Therapy (ATT) using the robot dog ‘AIBO’ (34). The participants received one hour of ATT once a week at the same time for two months (eight sessions in total). Activities included greeting and petting the robot, playing ball games, teaching AIBO to walk, performing daily living tasks, and taking pictures together. A psychiatrist assessed A-state STAI and PANSS scores pre- and post-intervention.

Results indicated improvement in PANSS scores across all three participants in at least one category, with no declines observed. A-state STAI scores also decreased in all participants. The findings suggest that ATT with AIBO may alleviate negative and general psychopathological symptoms in individuals with schizophrenia.

#### 3.2.2 Autism spectrum disorder

Kumazaki et al. (23) assessed the feasibility and preliminary effectivity of a robot-mediated mock job interview training for young adults with ASD. The study aimed to asses improvements in self-confidence and reductions in biological stress levels (saliva cortisol) compared to a psycho-educational approach. This randomised study included 15 young adults (ages 18-25) with a confirmed diagnosis of ASD based on the DMS-5 criteria, who were actively seeking employment and scored over 30 on the Liebowitz Social Anxiety Scale (LSAS) (35).

Participants were asked to choose a hypothetical job to apply for as part of the training before being randomised into the robot training group (n = 7) or the control group (n = 8). All participants underwent a ten-minute mock job interview with a human interviewer on days 1 and 5. During days 2-4, the robot group participated in similar mock job interviews mediated by ‘Actroid-F’ (36), a remotely controlled female humanoid robot with a highly human-like appearance (37). Meanwhile, the control group engaged in independent study by reviewing frequently asked interview questions for at least ten minutes daily.

After each human interview, participants rated their self-confidence on a Likert Rating Scale from 0-6 (0 = not at all comfortable, 6 = very comfortable). To measure biological stress levels, saliva samples were collected after human interviews and simultaneously on all other days. A two-way repeated measures analysis of variance (ANOVA) was used to examine the independent variables: ‘group’ (robot training vs. independent study) and ‘time’ (pre-/post-intervention).

A significant interaction effect between group and time was detected for saliva cortisol levels *(F = 2.63; p = 0.045*). The interaction effect approached significance between group and time for self-confidence ratings (*F = 2.24, p = 0.078*). A significant increase (F = 2.236, *p = 0.04*) in salivary cortisol was observed on day two compared to day one in the android-robot-mediated group, suggesting an initial enhancement of arousal. No significant changes were observed on other days. This transient cortisol increase may reflect a necessary physiological response to facilitate optimal performance during initial social interactions with new individuals, aligning with previous findings (38).

In summary, the study provides preliminary evidence that robot-mediated systems may be acceptable and feasible, with implications for real-world application.

Kumazaki et al. (24) performed another randomised controlled trial to evaluate the effectiveness of job interview training using the Actroid-F robot for young adults with ASD (ages 18-27). The study focused on the acquisition of nonverbal communication, self-reported self-confidence ratings, and biological metrics of stress (salivary cortisol levels) in 29 individuals actively seeking employment. Despite participating in successive mock job interviews (MJIs) prior to the study, these individuals had not improved their nonverbal communication skills.

Participants were required to meet diagnostic criteria for ASD according to the ‘Diagnostic Interview for Social and Communication Disorders (DISCO)’ (39), have an IQ of 70 or more, measured by the ‘Wechsler Adult Intelligence Scale - Fourth Edition’ (40), and a social anxiety score of at least 30 on the ‘Liebowitz Social Anxiety Scale (LSAS)’ (35).

Participants selected a job to apply for and were randomised into the intervention group (N = 13) or control group (N = 16). Both groups underwent mock job interviews with a human interviewer on days 1 and 7. From day 2 to day 6, the intervention group received job interview training using an android robot (abbreviated as ‘JUA’) and teacher guidance (named ‘IGT’), while the control group received IGT only.

The JUA was structured in three stages: (1) tele-operating the android robot to interact with others, (2) a face-to-face mock job interview with the android robot, and (3) feedback based on the mock job interview and a nonverbal communication exercise provided by the android robot. After the MJIs on day 1 and 7, all participants rated their self-confidence on a 7-point Likert-type scale (0 = not at all comfortable; 6 = very comfortable) and provided saliva samples at four measure points: S1 (baseline, right before MJI), S2 (immediately after MJI), S3 (20 min after MJI) and S4 (40 min after MJI). Two independent reviewers scored the nonverbal communication performance on a Likert Scale (0 = very poor; 6 = very excellent). An ANOVA was performed. In terms of nonverbal communication, significant improvements in posture (*F = 18.56; df = 1.23; p < 0.001; η^2^ = 0.447*), gaze (*F = 6.89; df = 1.23; p < 0.001; η^2^ = 0.750*), voice volume (*F = 13.64; df = 1.23; p < 0.001; η^2^ = 0.372*), nodding (*F = 70.01; df = 1.23; p < 0.001; η^2^ = 0.753*) and facial expressions (*F = 59.62; df = 1.23; p < 0.001; η^2^ = 0.722*) were observed in the combined group compared to the control. Self-reported self-confidence also improved significantly more in the combined group (*F = 5.67; df = 1.23; p = 0.026; η^2^=0.198*).

Regarding salivary cortisol, a greater rate of improvement was observed in the intervention group between S4/S1 and S3/S1, though no significant improvement was detected between S2/S1. These findings suggest that the tele-operating method may enhance the understanding of nonverbal communication and contribute to skill acquisition. Self-reported self-confidence also showed significant improvement, highlighting the potential effectiveness of robot-mediated job interview training for individuals with ASD.

#### 3.2.3 Intellectual disability

Shukla et al. (25) evaluated the response to robot interactions among six individuals with ID, focusing on engagement rates and changes in disability behaviour during interactions with the humanoid robot ‘NAO’ (41) compared to everyday situations. Participants were adults with an official diagnosis of ID who had lived for at least three years in ‘FAM’ (Ave Maria Foundation), a Spanish residential care facility for individuals with ID. All participants were already familiar with the NAO robot.

Interactive activities were designed according to four categories: entertainment (‘Dance Choreography’), physical training (‘Touch my head’), emotional adaptation treatment (‘Guess emotions’), and teaching (‘Learn the senses’). These activities were pre-programmed into NAO. For each session, the robot was placed on a table in front of the participant, with only the caregiver present to address any questions. Interventions lasted 15-30 minutes, with 5-10 minutes dedicated to robot interactions.

Engagement rates were calculated using video recordings. Disability behaviour was evaluated during everyday life situations, such as interacting with another resident, using a questionnaire adapted from the Gilliam Autism Rating Scale (GARS-2) (42), the Disability Assessment Schedule (WHODAS9) (43), and the Adaptive Behavior Scale: Residential and Community (ABS-RC:2) (44). It was then re-evaluated during the robot interaction. The results showed that the minimum engagement in non-interactive activities was 64.56% and 100% in interactive activities, indicating the greater appeal of interactive tasks. One caregiver evaluated disability behaviour based on the questionnaires and found a reduction in disability behaviour during the robot interactions compared to baseline scenarios. Informal observations also highlighted distinct behavioural differences based on participants’ varying ID levels, emphasising the need for customised applications. Higher disability leads to lower engagement; therefore, more attractive designs are required.

The study concluded that humans remain easier to follow than robots, but robots can be effective in capturing and sustaining attention. However, the potential side effects of robot interventions remain unclear, and adverse outcomes are possible. Specific staff training and standardised evaluation scales for robotic interventions were recommended for future applications.

Wagemaker et al. (26) explored the efficacy of the robot seal ‘Paro’ (45) in improving mood and alertness among five adults with varying levels of ID, comparing its impact to that of a plush toy seal. Participants were members of the same group at a residential mental healthcare institution.

The study was divided into two phases, each lasting four weeks. During the control phase, a plush seal (Tobi), designed to resemble Paro, was present in the living room. During the treatment phase, it was replaced with Paro. Both phases adhered to a structured protocol for presenting Tobi and Paro to participants, including daily interaction rituals. Additionally, participants were free to interact with the seal at their discretion. Throughout the eight-week study, mood and alertness ratings were provided twice a day by the daily supervisors (morning and evening shifts) using the ‘Young Child Session Rating Scale’ (46), and the ‘Alertness Observation List’ (47). Self-reported mood ratings were provided once a day before bedtime by pointing to one of three smileys (Young Child Session Rating Scale).

However, 17,6% of the data was missing, mainly due to incomplete self-reports. Results showed a positive influence of Paro on mood and alertness compared to the plush seal in only one participant, with no significant advantages observed for the other four. It was concluded that positive interactions with an animal-like robot could still have therapeutic value for some individuals, despite not seeing improvements in the selected outcome measures. However, mental health care institutions should exercise caution when investing in expensive therapeutic robot seals, as their effectiveness has not been proven.

### 3.3 Acceptance & perception

Only one of the included studies (24) analysed the acceptance of robot interventions in a structured and reproducible manner. However, further four studies provided interview quotes and informal observations.

Betriana et al. (20) assessed Pepper’s acceptance and perception through interviews after conversations. Both patients replied that they enjoyed talking to Pepper. Patient A said: “There was no problem with the conversation”. Patient B said: “I want to talk to Pepper again if I have the opportunity.” She was delighted she could talk to Pepper.

Cohen et al. (21) didn’t measure acceptance of their intervention.

Narita et al. (22) provided some quotes from the participants that gave clues on their perception of robot therapy. Case 1: “The first time, I did not like playing with the robot because I was depressed and anxious. But I feel good while playing with AIBO now. It heals my mind.” Case 2: “I’m looking forward to the next AIBO-assisted therapy session. After I played with AIBO, I felt good. I enjoy it.” Case 3: “After I played with AIBO, I felt good. I enjoy it with other patients.”

Kumazaki et al. (23) acknowledged that all participants completed the trial without relevant distress or technological issues that would lead to dropout. They confirmed that all participants were concentrating during the trials and were highly motivated from the start to the end of the experiment. This conclusion was derived from careful observation of the performance.

Kumazaki et al. (24) asked all participants, “Do you want to receive this intervention again?” on day six of the experiment. In the combined group (including android-robot mediated training), 100% of the participants answered “Yes,” and everyone completed the trial. In the control group, only 53.8% said they wanted to receive the intervention again. Three participants dropped out of the study because they struggled to maintain motivation. Shukla et al. (25) didn’t measure acceptance. Wagemaker et al. (26) observed that Paro drew more attention than the plush seal. Mild anxiety was noted in some participants at the introduction of Paro because of its unpredictable sounds and movements, but this quickly diminished. The caretakers thought PARO was especially useful for Participant 2, although they did not improve on the chosen outcome measures.

### 3.4 Quality evaluation

The results of the quality assessment are summarised in **Table 2**.

**Table 2:** Criteria from the Mixed Methods Appraisal Tool.

Studies	1.1	1.2	1.3.	1.4.	1.5.	2.1.	2.2.	2.3.	2.4.	2.5	3.1.	3.2.	3.3.	3.4.	3.5.
1) Betriana et al. (20)	1	1	–	–	–										
2) Cohen et al. (21)											1	1	1	1	1
3) Narita et al. (22)	1	1	–	1	1										
4) Kumazaki et al. (23)						–	1	1	–	1					
5) Kumazaki et al. (24)						–	1	1	0	1					
6) Shukla et al. (25)	1	1	1	1	1										
7) Wagemaker et al. (26)	1	1	1	1	1										
1= Yes; 0= No; - = Can’t tell															
1.1 Is the qualitative approach appropriate to answer the research question?															
1.2 Are the qualitative data collection methods adequate to address the research question?															
1.3 Are the findings adequately derived from the data?															
1.4 Is the interpretation of results sufficiently sustained by data?															
1.5 Is there coherence between qualitative data sources, collection, analysis and interpretation?															
2.1 Is randomization appropriately performed?															
2.2 Are the groups comparable at baseline?															
2.3 Are there complete outcome data?															
2.4 Are outcome assessors blinded to the intervention provided?															
2.5 Did the participants adhere to the assigned intervention?															
3.1 Are the participants representative of the target population?															
3.2 Are measurements appropriate regarding both the outcome and the intervention (or exposure)?															
3.3 Are there complete outcome data?															
3.4 Are the confounders accounted for in the design and analysis?															
3.5 During the study period, is the intervention administered (or exposure occurred) as intended?															

Four out of seven studies (20, 21, 23, 24) showed some quality deficits, mainly because the information necessary to make a judgment was missing. The main complaint regarding the two RCTs (23, 24) was a need for more information on randomisation procedures, and it was disclosed that at least some of the researchers who measured target outcomes were not blinded to the group assignment. We must assume that researcher bias may have influenced the results. In the case of the study (20), we agreed that the qualitative approach was adequate to answer the research question and that data was appropriately collected. However, coherence between data sources, their collection, and interpretation was not proven to be coherent and sufficiently substantiated by data. (22) only lacked information regarding data derivation.

## 4 Discussion

### 4.1 Summary

#### 4.1.1 Target variables

This scoping review aims to provide an overview of current research on using social robots in adult psychiatry.

The included studies (N = 7) mainly consisted of qualitative research with small sample sizes (3-44 participants) and loosely defined target measures. The target variables included mood and alertness, engagement, disability behaviour, self-confidence, salivary cortisol levels, positive and negative symptoms of schizophrenia, anxiety scores, the influence of social feedback, and communication characteristics. The roles of the social robots during the interventions can be summarised into three groups: a companion and entertainer (N = 3), a teacher/instructor (N = 3), and a communication partner (N = 3). The robots were either humanoid (N = 5) or zoomorphic (N = 2). The robot could fulfil more than one role at a time.

#### 4.1.2 Observations, effects & acceptance

Although the effects were diverse, the overall trend was promising. Betriana et al. (20) found that the robot interaction was enjoyable for the participants, a two-way conversation was possible, and an intentional dialogue was initiated. Cohen et al. (21) showed that the ability of schizophrenia patients to synchronise with a robot partner seems to be impaired. Narita et al. (22) showed that the pet robot AIBO might be helpful for patients with schizophrenia suffering from negative and general symptoms. Kumazaki et al. (23, 24) concluded that robot-mediated systems might be feasible and acceptable for young adults with ASD to improve their mock-job interview skills, including verbal and non-verbal communication, and might help boost their self-confidence and reduce stress in this social situation. Shukla et al. (25) noted the reduction of disability behaviour in people with ID and stressed the importance of interactive, customised applications to draw attention. Wagemaker et al. (26) concluded that positive interactions with the robot might be a therapeutic aim in themselves despite not seeing improvement in their chosen outcome measures. They argued that the mood improvement of some individuals during the session and emotional connections towards the robot animal are valuable, nonetheless.

#### 4.1.3 Quality and limitations

None of the studies offered a holistic analysis that could objectively assess acceptance of the interventions and robots. We can assume that the interventions were enjoyable and beneficial to at least some participants. The reported distress and dropout rates were minimal. According to the MMAT, quality showed room for improvement, but the overall quality was acceptable.

The experimental design’s limitations need to be considered. Included studies were primarily based on qualitative observations, often unstructured and difficult to reproduce. Five out of seven could be classified as case reports or pilot studies. No study used a power analysis to ensure the statistical significance of the findings. The results of the two RCTs could have been distorted by selection and researcher biases.

### 4.2 Consequences for future research

Although no universal advice can be derived, it is promising to see some positive effects and reactions to robot therapy. The synopsis of results demonstrates the diversity of possible applications of social robots in adult psychiatry and could serve as an inspiration. Future researchers should aim to pick up where others left off, building upon the insights and addressing the limitations of the studies presented in this paper. Regarding robot therapy designs, the review revealed the importance of interactive features, some level of autonomic functioning, and appropriate guidance from a human facilitator. The target group’s specific needs should be kept in mind. The robot’s range of abilities should neither be overwhelming nor disappointing to the participants. Sometimes, individual customisation might be necessary to achieve satisfactory results. To gain meaningful insight into social robots in the context of psychiatry, participants need to be selected under clear inclusion criteria regarding their mental health history and officially recognised diagnoses. It would be valuable to explore the benefits for individuals with chronic mental conditions as well as those who are generally healthy but experience occasional mental distress. Given the positive trend but low statistical power of existing studies, further trials should include larger sample sizes and narrowly defined target variables. More quantitative, replicable research would be highly valuable. Demand for comprehensive studies focused on acceptability remains as well.

### 4.3 Implementation

Given the absence of conclusive data to recommend established therapies or preferred applications, there remains considerable potential for exploring the integration of social robots into facility-based mental health care.

Rasouli et al. (9) discussed various roles a social robot could assume in clinical interventions for individuals with social anxiety disorder. The list includes robot-mediated interviews, robot-assisted therapy, social robots as diagnostic agents or interactive social companions, as playmates or social mediators, as coaches and instructors. Although this list is neither exhaustive nor universally transferable, it can inspire methods of integrating social robots into psychiatric care.

Further examples include: social robots as a motivational coach to encourage the reduction of high-calorie foods and drinks between meals (8) and as a screening tool to detect signs of post-traumatic stress disorder in trauma survivors right in the emergency department (48).

### 4.4 Social robot classification

Building upon the various roles of social robots discussed in the previous section, we propose that the most efficient method of categorising social robots is based on their functional roles. Within the context of psychiatric care and therapy, three primary roles have been identified: Therapist/Coach (a new medium of delivering psychotherapy), Mediator (a facilitator of treatment) and Assistant (involved in patient assessment and skill training) (9). Based on our review, we concur that this categorisation is meaningful due to its focus on both experience and outcomes. However, we suggest that the role ‘Companion’ could serve as a valuable addition to this framework. Alleviating feelings of loneliness, which are often associated with depression, represents a therapeutic goal that social robots could address to some extent (11). The robots discussed in this paper predominantly align with the roles of ‘Coach’ and ‘Companion’.

### 4.5 Limitations of this paper

This review has several limitations. First, it is restricted to articles indexed in PubMed and PsycINFO, as well as those identified through citation screening and informal searches. Despite efforts to ensure thoroughness, it is possible that some eligible studies were overlooked. Due to the diverse nature of the identified studies, it was not feasible to provide a more structured summary of target variables and effects; therefore, a narrative approach was employed to offer a general overview. Studies involving both children and adults were excluded if their results could not be disaggregated. This review does not encompass ongoing, unpublished projects or studies published in languages other than English.

### 4.6 Considerations and chances

Implementing new technologies and lowering staff workload should not result in the replacement of established services or human professionals. Fiske et al. (49) highlighted several ethical considerations in their review titled ‘Your Robot Therapist Will See You Now.’ While raising concerns about issues such as data ethics and the potential for misuse, the authors also identified several benefits, including the opportunity to free up healthcare staff time for more impactful tasks, the introduction of new therapeutic approaches, improved patient satisfaction, and the possibility of reaching previously underserved populations.

With these considerations in mind, we should remain open and curious about future advances in robotics and the opportunities they may offer. To enhance access to mental health care, Laban et al. (48) proposed that humanoid robots, such as ‘Pepper’ or ‘NAO’, could be placed in people’s homes or other familiar spaces to routinely collect data on the residents’ mental well-being. In doing so, they could screen for mental health issues in a natural, conversational manner and offer early interventions, with individuals then referred to a human mental health professional as needed. Furthermore, robots like Pepper could serve as a telepresence tool, remotely controlled by clinicians, to deliver psychotherapy to people’s homes in a more engaging and accessible way.

### 4.7 Conclusion

In conclusion, social robots in psychiatry should be viewed as a chance to expand current mental health treatment and care mindfully and holistically. Considering human resources, financial limits, and social barriers, we should work towards making good mental health care a standard for everybody.

## Data Availability

The original contributions presented in the study are included in the article/supplementary material. Further inquiries can be directed to the corresponding author.
